# Predictors of cigarette smoking frequency among European adolescents aged 13–15: the critical role of parental smoking and age of initiation

**DOI:** 10.1007/s00787-025-02772-z

**Published:** 2025-06-06

**Authors:** Omid Dadras, Anne Abio

**Affiliations:** 1 Research Center for Child Psychiatry, University of Turku, Lemminkäisenkatu 3, Turku, 20014 Finland; 2 INVEST Research Flagship Center, University of Turku, Turku, Finland

**Keywords:** Cigarette, Tobacco, Adolescent, Europe, Predictor

## Abstract

This cross-sectional study investigated predictors of cigarette smoking frequency among European adolescents aged 13–15, focusing on parental smoking, age of initiation, and socioeconomic factors. Data were derived from the Global Youth Tobacco Survey (2020–2023) in eight European countries. Smoking frequency was classified as infrequent (< 1/day), daily light (1/day), moderate (2–5/day), and heavy (> 5/day). Individual variables (age, pocket money, age at initiation, tobacco experimentation), familial factors (parental smoking, parental education), and country-level factors (PPP-adjusted cigarette prices, income inequality) were analyzed using sex-stratified multilevel ordinal logistic regression models, accounting for clustering at the country and school levels. Notable country- and gender-specific variations in smoking prevalence were observed. Notably, Bulgaria and Albania exhibited the highest prevalence of heavy smoking (> 5 cigarettes/day). Overall, girls were more likely to be smokers, while boys tended to be heavy smokers. Older age, early initiation (< 10 years), and tobacco experimentation significantly increased smoking frequency in both sexes. For males, paternal smoking predicted higher frequency (OR = 2.06), whereas maternal smoking appeared protective (OR = 0.67). Among females, maternal smoking and dual parental smoking were associated with increased frequency. Higher pocket money was also associated with smoking frequency, while cigarette affordability showed a marginal inverse association in males. Our findings underscore the critical role of early smoking initiation and parental influence in determining smoking frequency among European adolescents. Tailored interventions addressing familial risk factors and socioeconomic determinants are essential to curb heavy smoking in this vulnerable population.

## Introduction

Adolescent smoking remains a critical public health challenge across Europe despite decades of tobacco control policies. While overall smoking rates have declined in recent years, the persistent initiation of tobacco use during early adolescence continues to raise significant concerns. Currently, approximately 15% of young people aged 15–24 in the European Union smoke daily, with notable gender disparities (18% of young men and 12% of young women) [1]. The transition from experimentation to regular smoking frequently occurs during early adolescence, with over half of young smokers establishing regular patterns by age 18 [2]. This early initiation is particularly concerning given that nicotine is among the most addictive substances known, with tobacco use beginning in adolescence often developing into serious addiction with lasting health consequences [3].

The epidemiological landscape of adolescent smoking in Europe presents a complex picture characterized by both encouraging trends and persistent challenges. While EU-overall smoking initiation rates among those aged 10–24 have significantly declined over recent decades (from 5.7% in the 1970s to 3.2% in the 2010s for males, and from 3.9% in the 1990s to 2.4% in the 2010s for females), substantial regional variations persist [2]. Countries such as Hungary (26%) and Austria (19%) continue to report alarmingly high youth smoking rates, while Sweden (5%) and Finland (7%) demonstrate considerably lower prevalence [4]. Of particular concern is the observation that the decline in smoking initiation has been less pronounced among legal minors aged 10–17 compared to young adults, with minors’ initiation rates actually surpassing those of young adults during the 2010s in some regions [2].

Existing literature on adolescent smoking behaviors has established several key insights regarding prevalence and risk factors. Cross-sectional studies have consistently identified socioeconomic status as a significant predictor of smoking behavior, with considerable differences in daily smoking according to educational track observed across multiple European countries [5]. These socioeconomic inequalities appear to be widening in some contexts, with absolute educational disparities dramatically increasing in countries like Croatia and Italy [5]. Gender differences in smoking prevalence have also been extensively documented, though the traditional gap between male and female smoking rates is rapidly narrowing, with girls equaling or surpassing boys in rates of smoking by age 15 in some countries [6]. Yet, the mechanisms underlying these trends—including how parental smoking (e.g., same-sex parent influence) and socioeconomic factors (e.g., pocket money) interact to shape smoking frequency—are poorly understood.

To guide this study, we drew on two complementary theoretical frameworks: Social Learning Theory [7] and Problem Behavior Theory [8]. Social Learning Theory posits that adolescents acquire behaviors such as smoking through observation, imitation, and reinforcement within their social environment, particularly from significant role models like parents and peers [9]. Problem Behavior Theory expands on this by situating adolescent smoking within a broader constellation of risk behaviors influenced by personality traits, perceived environmental controls, and socioeconomic contexts [10]. Based on these frameworks, we hypothesized that (H1) parental smoking, especially by the same-sex parent, would increase the likelihood of more frequent adolescent smoking; (H2) early smoking initiation would be associated with higher smoking frequency; (H3) greater access to pocket money would predict higher smoking frequency due to increased purchasing power; and (H4) country-level factors such as cigarette affordability would inversely relate to smoking frequency. Together, these hypotheses reflect the interplay between individual, familial, and environmental determinants shaping adolescent smoking behaviors in Europe.

This study addressed these hypotheses by analyzing data from the Global Youth Tobacco Survey (GYTS) across eight European countries (2020–2023). Specifically, we aimed to examine country- and sex-specific variations in cigarette smoking frequency among adolescents aged 13–15, identify gender-stratified predictors, including parental smoking dynamics (e.g., same-sex parent roles), socioeconomic factors (e.g., pocket money), and age of initiation, and assess the influence of macro-level factors, such as cigarette affordability and income inequality, on smoking behaviors. By elucidating these pathways, this research informs tailored tobacco control strategies that account for regional, socioeconomic, and gender-specific vulnerabilities, with a focus on mitigating progression to frequent and heavy smoking.

## Methods

### Data source

This cross-sectional study utilized data from the Global Youth Tobacco Survey (GYTS), a nationally representative school-based survey targeting adolescents aged 13–15 years. We included all European countries for which the GYTS wave was conducted between 2020 and 2023. This resulted in eight countries: Albania (2020), Belarus (2021), Bulgaria (2023), Czechia (2022), Italy (2022), Lithuania (2022), Poland (2022), and San Marino (2022). The GYTS employs a standardized two-stage cluster sampling methodology, with schools as primary sampling units and classrooms as secondary units, ensuring geographic and demographic representativeness. Ethical approvals were obtained by coordinating institutions in each country as part of the GYTS protocol, with written informed consent from participants. Further information about the study design and sampling can be found elsewhere [11]. Country-specific response rates were: Albania (90.4%), Belarus (81.2%), Bulgaria (79.5%), Czechia (62.0%), Italy (52.5%), Lithuania (74.0%), Poland (74.8%), and San Marino (92.1%).

### Study variables

The study variables were selected based on a comprehensive literature review and the availability of relevant variables in the GYTS dataset.

### Outcome variables

The main outcome variable was cigarette smoking frequency, categorized as: <1/day (infrequent), 1/day (daily light), 2–5/day (moderate), and > 5/day (heavy) and measured among those who smoked cigarettes in the past month.

### Explanatory variables

#### Individual factors

Age (13, 14, 15 years); sex (self-reported by respondents as male or female and treated as a binary biological variable in all analyses); pocket money (harmonized into country-specific terciles: low, medium, high-based, on national distributions); age at smoking initiation, defined as the age of first cigarette use and categorized as < 10, 10–11, 12–13, or 14–15 years; beliefs about tobacco risks (dangerous vs. not dangerous) and perceived difficulty of quitting (difficult vs. not difficult); and tobacco experimentation (tried other tobacco products: yes/no).

#### Family- and friends-related factors

Parental smoking status (none, father only, mother only, both parents), self-reported by adolescents; parental education (primary, secondary, higher) for both mothers and fathers; peer smoking (close friends smoke: yes/no).

#### Country-level factors

Cigarette price (PPP-adjusted) plays a crucial role in smoking behavior, as affordability influences both initiation and frequency. It was calculated using the formula: Price of Cigarette / PPP conversion factor. Country-specific nominal cigarette prices were manually assigned based on survey-year data, while the corresponding PPP conversion factors were obtained from the World Bank. The PPP conversion factor accounts for differences in the relative value of local currencies compared to international dollars, enabling a more accurate cross-country comparison of cigarette prices.

### Statistical analysis

Descriptive statistics were used to estimate the prevalence of cigarette smoking and its frequency distribution across the included countries by sex. Sex-stratified multilevel ordinal logistic regression models, accounting for clustering at the country and school levels, were used to examine the association between individuals and family/friend-related factors with cigarette smoking frequency. To account for the complex survey design and ensure representativeness, survey weights were incorporated into the analysis. The original sampling weight variable, provided in the GYTS dataset, was adjusted to align with the study’s specific analytical requirements. First, the total sum of sample weights was calculated and used to rescale individual weights proportionally to the observed sample size of the 13–15-year-old population in each country. Specifically, the rescaled weight was generated by dividing the country-specific sample size of adolescents aged 13–15 by the total sum of sample weights. Finally, standardized weights were computed as the product of the original weight and the rescaling factor. This adjustment ensured that the weighted sample size matched the actual study population, preventing inflation of statistical power while preserving the representativeness of the clustered sampling framework.

Although our analysis includes a relatively small number of level-3 units (*n* = 8 countries), we opted for a three-level multilevel ordinal logistic regression to properly account for clustering at both the school and country levels. Simulation studies indicate that, while variance-component estimates may be less precise with few clusters, fixed‐effect estimates remain unbiased even with as few as five higher-level units [12]. Moreover, the observed country-level intraclass correlation coefficients (0.07–0.16) underscore non‐negligible between-country heterogeneity that, if ignored, could lead to understated standard errors and inflated Type I error rates. Because our primary inferences concern individual- and family-level predictors, incorporating country-level random intercepts ensures valid estimate precision and inference. Model 1 (null model) estimated with no covariates included estimates the clustering effect by calculating Intraclass correlation coefficients (ICC), indicating meaningful clustering (country ICC: 0.08–0.11; school ICC: 0.18–0.19), justifying multilevel modeling. Model 2 was adjusted for individual factors (age, pocket money, age at initiation, beliefs about tobacco, quitting difficulty, and tobacco experimentation). Model 3 further incorporated family- and peer-level variables (parental smoking, parental education, peer smoking). In both models 1 and 2, country-level economic indicators, including cigarette prices (PPP-adjusted) and Gini coefficients, were included to account for the disparity in income inequality and cost of living.

Missing data (< 5% for all variables) were handled via listwise deletion. Analyses were conducted in Stata/SE 17.0. To assess the proportional odds assumption, we performed a Brant test on a comparable single-level ordinal logistic model; no significant violations were detected (all *p* > 0.10). We confirmed multicollinearity was not problematic (all VIFs < 2.5). Random intercepts at the school and country levels were inspected via Q-Q plots and appeared approximately normally distributed. As a sensitivity check, we conducted a backward elimination stepwise procedure (entry/removal at α = 0.05), which retained the same core predictors (age, age at initiation, pocket money, parental smoking, cigarette affordability) and yielded comparable AIC/BIC and odds ratios, supporting the robustness of our theory-driven model.

**Fig. 1:**
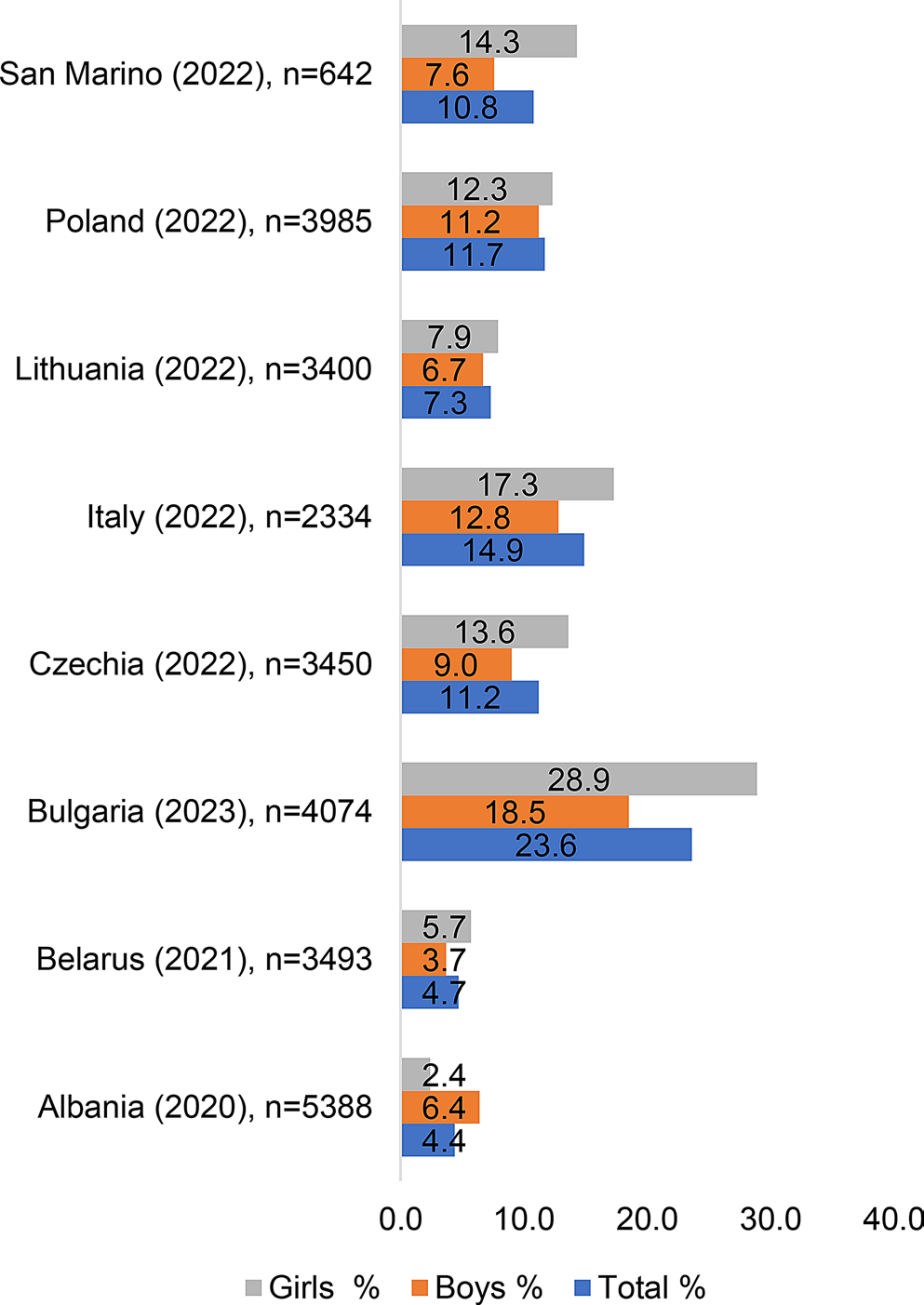
The prevalence of cigarette smoking among adolescents aged 13–15 years (GYTS 2020–2023)

## Results

### Prevalence and frequency of cigarette smoking

Figure 1 illustrates the overall prevalence of cigarette smoking among adolescents aged 13–15 across the eight European countries included in the study. Notable differences were observed by country and sex. For example, in Bulgaria (2023), the prevalence among females was substantially higher (28.9%) compared to males (18.5%). In contrast, countries such as Poland (2022) and Lithuania (2022) showed more balanced distributions between sexes.

As shown in Table 1, among adolescents who reported smoking in the past month, the distribution of smoking frequency varied significantly by country and sex. Bulgaria exhibited the highest proportion of heavy smokers (> 5 cigarettes/day) for both males (48.0%) and females (46.9%), while Albania demonstrated stark sex differences, with females reporting higher infrequent smoking (< 1/day: 39.7%) and males exhibiting higher heavy smoking (24.9%). Significant sex disparities in smoking frequency distributions were observed in Albania (*p* = 0.013) and Czechia (*p* = 0.039), but not in other countries (e.g., Bulgaria: *p* = 0.904; Poland: *p* = 0.139).

**Table 1:** Frequency distribution of cigarette smoking among adolescents aged 13–15 years who reported past-month smoking: by country and sex (GYTS 2020–2023)

Country (Year)	Sex	Frequency of cigarette smoking (%)				*p*-value*
		< 1/day	1/day	2–5/day	> 5/day	
Albania (2020)	Male	27.4	24.6	23.1	24.9	
	Female	39.7	35.3	21.6	3.5	0.013
Belarus (2021)	Male	31.8	26.4	23.7	18.2	
	Female	33.0	29.5	28.4	9.1	0.382
Bulgaria (2023)	Male	13.5	9.2	29.3	48.0	
	Female	11.4	10.4	31.3	46.9	0.904
Czechia (2022)	Male	35.7	23.7	34.5	6.1	
	Female	31.3	21.4	30.6	16.6	0.039
Italy (2022)	Male	37.5	22.9	28.5	11.1	
	Female	35.2	28.5	27.3	9.1	0.170
Lithuania (2022)	Male	44.1	18.5	22.7	14.8	
	Female	35.1	32.4	22.5	10.0	0.244
Poland (2022)	Male	23.7	20.9	35.1	20.3	
	Female	31.1	25.2	29.2	14.5	0.139
San Marino (2022)	Male	41.7	14.5	21.9	22.0	
	Female	28.9	22.3	38.0	10.8	0.385

* p-values based on Pearson’s chi-square tests for differences in frequency distributions by sex

### Factors associated with smoking frequency

Multilevel ordinal logistic regression models (Tables 2 and 3) revealed distinct predictors of smoking frequency for males and females after adjusting for individual, familial, and country-level factors.

### Individual factors

For males, older age (15 vs. 13 years) was associated with higher smoking frequency in Model 2 (OR = 3.33, 95% CI: 2.23–4.96), but this association attenuated in Model 3 (OR = 2.23, 95% CI: 0.87–5.75). In contrast, females aged 15 had persistently higher odds of frequent smoking even after full adjustment (OR = 3.52, 95% CI: 1.67–7.43). High pocket money was associated with higher smoking frequency for males (Model 3: OR = 1.82, 95% CI: 1.09–3.05) and females (Model 3: OR = 1.46, 95% CI: 0.76–2.81), though the latter was not statistically significant. Earlier initiation (< 10 years vs. 14–15 years) strongly predicted higher smoking frequency for both sexes (Males: OR = 0.11, 95% CI: 0.05–0.25; Females: OR = 0.21, 95% CI: 0.08–0.56). Adolescents who tried other tobacco products had significantly higher odds of frequent smoking (Males: OR = 1.96, 95% CI: 1.05–3.63; Females: OR = 2.98, 95% CI: 2.04–4.33).

### Family and peer factors

For males, paternal smoking alone was associated with higher smoking frequency (OR = 2.06, 95% CI: 1.26–3.38, *p* < 0.01), and in contrast, maternal smoking was associated with lower smoking frequency (OR = 0.67, 95% CI: 0.48–0.93). Among females, having both parents smoke increased the odds (OR = 1.58, 95% CI: 1.12–2.24), as did maternal smoking (OR = 1.34, 95% CI: 1.01–1.79), but not paternal smoking. Close friends’ smoking was not statistically significant in fully adjusted models for either sex (Males: OR = 0.92, 95% CI: 0.38–2.26; Females: OR = 1.44, 95% CI: 0.71–3.04).

### Country level factors

While the cigarette price (PPP-adjusted) was associated with a lower likelihood in both males and females, it was only significant in Model 2 for males (OR = 0.71, 95% CI: 0.51–0.98).

### Model fit and clustering effects

Both models were evaluated using AIC and BIC criteria, which indicated a good model fit. Intraclass correlation coefficients (ICCs) demonstrated significant clustering effects, with country-level ICCs ranging from 0.07 to 0.16 and school-level ICCs between 0.18 and 0.19, justifying the use of multilevel modeling.

**Table 2:** Factors associated with cigarette smoking frequency among adolescents aged 13–15: males (GYTS 2020–2023)

	Frequency distribution of cigarette smoking (%)					Model 1 (null)	Model 2	Model 3
	< 1/day	1/day	2–5/day	> 5/day	*p*-value		OR (95% CI)	OR (95% CI)
Age (years)								
13	35.8	26.6	26.7	10.9		–	Ref.	Ref.
14	27.9	15.5	31.2	25.4		–	2.83 (1.73–4.62)*	2.02 (0.77–5.33)
15	25.1	18.9	28.4	27.6	< 0.001	–	3.33 (2.23–4.96)*	2.23 (0.87–5.75)
Pocket money								
Low	32.5	24.2	26.5	16.9		–	Ref.	Ref.
Medium	33.5	19.8	29.1	17.7		–	0.92 (0.69–1.22)	1.02 (0.58–1.80)
High	22.8	16.7	30.1	30.4	< 0.001	–	1.54 (1.19–1.99)*	1.82 (1.09–3.05)*
Age at initiation								
< 10	12.8	15.3	30	41.8		–	Ref.	Ref.
10-11	25.2	15.3	37.7	21.8		–	0.50 (0.33–0.75)*	0.33 (0.13–0.82)*
12-13	29.4	21.3	28.6	20.7		–	0.43 (0.35–0.53)*	0.36 (0.14–0.89)*
14-15	39.8	20	24	16.3	< 0.001	–	0.22 (0.13–0.35)*	0.11 (0.05–0.25)*
Tried other tobacco								
No	44.2	21.3	24.1	10.4		–	Ref.	Ref.
Yes	21.4	17.9	31.4	29.2	< 0.001	–	2.84 (2.29–3.51)*	1.96 (1.05–3.63)*
Believe tobacco is dangerous								
No	23.1	14.1	25	37.9		–	Ref.	Ref.
Yes	29.8	21.5	31.4	17.3	< 0.001	–	0.58 (0.43–0.80)*	0.79 (0.33–1.89)
Believe quitting is difficult								
No	29.8	18.1	30.5	21.6		–	Ref.	Ref.
Yes	28.2	20.1	28.8	22.9	0.832	–	1.35 (1.21–1.51)*	1.08 (0.66–1.77)
Parents smoke								
No	36.8	20.6	26.6	15.9		–	Ref.	Ref.
Both	20.1	17.5	36.1	26.2		–	–	1.47 (0.96–2.25)
Father only	25.9	20.8	26	27.2		–	–	2.06 (1.26–3.38)*
Mother only	33	17.4	26.5	23.1	< 0.001	–	–	0.67 (0.48–0.93)*
Mother education								
Primary	33.2	19.1	23.6	24.1		–	Ref.	Ref.
Secondary	21.7	25.8	31	21.4		–	–	1.39 (0.34–5.75)
Higher	25.9	16.3	28.5	29.3	0.07	–	–	1.19 (0.41–3.42)
Father education								
Primary	30.2	16.1	29	24.7		–	Ref.	Ref.
Secondary	23	24.9	29.3	22.8		–	–	1.04 (0.35–3.13)
Higher	26.6	16.7	27	29.8	0.226	–	–	1.19 (0.28–4.96)
Close friend smoke								
No	30.9	24.4	23.1	21.7		–	Ref.	Ref.
Yes	18.7	15.4	35.3	30.6	0.001	–	–	0.92 (0.38–2.26)
Cigarette prices (PPP-adjusted)	–	–	–	–		–	0.71 (0.51–0.98)*	0.88 (0.07–10.97)
AIC	–	–	–	–		2726.27	2178.56	713.46
BIC	–	–	–	–		2751.23	2212.52	728.59
ICC (country)	–	–	–	–		0.08	0.07	0.15
ICC (school| country)	–	–	–	–		0.18	0.19	0.19

* p-value > 0.05

**Table 3:** Factors associated with cigarette smoking frequency among adolescents aged 13–15: females (GYTS 2020–2023)

	Frequency distribution of cigarette smoking (%)					Model 1 (null)	Model 2	Model 3
	< 1/day	1/day	2–5/day	> 5/day	p-value		OR (95% CI)	OR (95% CI)
Age (years)								
13	32	25.7	32.8	9.6		–	Ref.	Ref.
14	27.9	21.9	30.1	20.1		–	1.77 (1.41–2.22)*	1.30 (0.97–1.76)
15	21.7	19.3	26.5	32.5	< 0.001	–	3.50 (2.75–4.45)*	3.52 (1.67–7.43)*
Pocket money								
Low	23.3	22.1	35.6	19		–	Ref.	Ref.
Medium	28.7	25.2	26.1	20		–	1.41 (1.12–1.78)*	1.30 (0.93–1.83)
High	25.6	18	27.9	28.6	0.116	–	1.65 (1.20–2.27)*	1.46 (0.76–2.81)
Age at initiation								
< 10	16	14.6	34.2	35.2		–	Ref.	Ref.
10-11	18.1	20.3	32.5	29		–	0.67 (0.40–1.13)	0.64 (0.16–2.55)
12-13	24.8	20	32.5	22.6		–	0.42 (0.31–0.58)*	0.32 (0.13–0.81)*
14-15	35.4	25.9	19.3	19.3	< 0.001	–	0.24 (0.18–0.32)*	0.21 (0.08–0.56)*
Tried other tobacco								
No	36.8	26.6	23.7	12.9		–	Ref.	Ref.
Yes	18.5	17.5	32.7	31.3	< 0.001	–	2.47 (1.96–3.12)*	2.98 (2.04–4.35)*
Believe tobacco is dangerous								
No	20.2	21.1	31.5	27.2		–	Ref.	Ref.
Yes	27	21.8	28.9	22.4	0.451	–	0.69 (0.50–0.94)*	0.78 (0.31–1.99)
Believe quitting is difficult								
No	26	19.1	29.9	25		–	Ref.	Ref.
Yes	26.3	22.8	28.9	22	0.646	–	1.30 (1.09–1.56)*	1.09 (0.73–1.63)
Parents smoke								
No	33.9	24.6	30.6	10.9		–	Ref.	Ref.
Both	18	20.7	29.3	32.1		–	–	1.58 (1.12–2.24)*
Father only	27.2	24.2	24.5	24.1		–	–	1.01 (0.65–1.57)
Mother only	23.4	15.7	35.5	25.4	0.015	–	–	1.34 (1.01–1.79)*
Mother education								
Primary	26.4	25.6	35.4	12.6		–	Ref.	Ref.
Secondary	31.7	23.5	25.5	19.3		–	–	0.60 (0.20–1.83)
Higher	21	19	29.7	30.3	0.041	–	–	0.87 (0.25–3.00)
Father education								
Primary	35.2	20.7	29.4	14.6		–	Ref.	Ref.
Secondary	29.4	30.9	24.7	15		–	–	1.28 (0.81–2.04)
Higher	18.7	16.6	33.3	31.3	< 0.001	–	–	1.06 (0.54–2.06)
Close friend smoke								
No	42.7	26.2	19	12.1		–	Ref.	Ref.
Yes	14.7	17.4	33	34.9	< 0.001	–	–	1.44 (0.71–2.90)
Cigarette prices (PPP-adjusted)	–	–	–	–		–	0.77 (0.53–1.22)	0.23 (0.02–2.58)
AIC	–	–	–	–		3255.35	2743.05	917.64
BIC	–	–	–	–		3280.84	2777.98	933.22
ICC (country)	–	–	–	–		0.11	0.09	0.16
ICC (school| country)	–	–	–	–		0.19	0.18	0.19

* p-value > 0.05

## Discussion

This study examined the predictors of cigarette smoking frequency among European adolescents aged 13–15 using data from the Global Youth Tobacco Survey (GYTS) across eight European countries. Key findings revealed significant country- and sex-specific variations in smoking prevalence and frequency. Notably, Bulgaria and Albania exhibited the highest prevalence of heavy smoking (> 5 cigarettes/day), with stark sex differences in Albania (higher infrequent smoking among females vs. heavy smoking among males). Older age, an earlier age of initiation (< 10 years), and tobacco experimentation were strongly associated with increased smoking frequency in both sexes. The impact of parental smoking was nuanced and differed by the sex of the adolescent. For males, paternal smoking emerged as a significant predictor of higher smoking frequency, while maternal smoking was a protective factor. On the contrary, among females, maternal smoking was the most influential factor, along with both parents smoking. Peer smoking did not emerge as a significant predictor after adjusting for parental and individual-level factors. Additionally, higher levels of pocket money were linked to more frequent smoking. Cigarette affordability (PPP-adjusted prices) showed a marginal association with reduced smoking frequency in males.

The observed patterns across different sexes and countries showed females predominantly engaged in infrequent smoking while males were more likely to be heavy smokers. These patterns are consistent with broader European surveillance data, which indicate that cultural, economic, and policy differences contribute to national disparities in youth smoking behaviors [13–15]. For example, recent analyses by the European Commission emphasize that regional socioeconomic conditions and local tobacco control policies are critical determinants of smoking prevalence in adolescents [16]. Furthermore, gender-specific social norms may explain why male adolescents in certain countries are more inclined toward heavier smoking, whereas female adolescents might limit their intake due to differing social pressures and perceptions of risk [14, 17]. These insights also carry significant implications for public health strategies. In countries like Bulgaria, where heavy smoking is prevalent among both sexes, aggressive interventions such as stricter tobacco pricing policies, targeted cessation programs, and robust public education campaigns are essential. Conversely, in countries like Albania, where female adolescents are more likely to engage in occasional smoking, interventions could focus on preventing progression to heavy smoking by addressing early experimentation and modifying social influences.

Older age was strongly associated with higher smoking frequency, and early initiation (particularly before age 10) emerged as one of the most robust predictors of heavier smoking in both sexes. This aligns with previous research showing that early exposure to nicotine increases the likelihood of developing more entrenched smoking habits later in adolescence and adulthood [18, 19]. Nicotine exposure during adolescence alters brain development, particularly in reward pathways, leading to higher dependence, frequency, and reduced cessation success [20, 21]. Research has shown that older adults who initiated smoking early face greater challenges in quitting, though lighter smokers (< 10 cigarettes/day) have higher cessation rates [22]. Early initiation is linked to persistent smoking patterns, cardiovascular risks, and lower cessation success [23], underscoring the critical window of adolescence for smoking interventions, as early initiation strongly predicts entrenched smoking behaviors and health risks in adulthood. In addition to early initiation, tobacco experimentation significantly predicted increased smoking frequency across both sexes in our study. Adolescents who try other tobacco products are more likely to transition into regular cigarette use; a pattern that has been documented in several longitudinal studies [24, 25]. These findings underscore the need for early intervention programs that target not only cigarette use but also experimentation with alternative tobacco products.

Economic factors also played a noteworthy role. Adolescents with higher levels of pocket money were more likely to engage in frequent smoking. This finding is consistent with evidence that disposable income increases access to tobacco products, thus facilitating experimentation and habitual use [26, 27]. A previous study in Europe has shown that teens in the highest income quintile had significantly higher odds of being daily smokers than those in the lowest quintile [28]. Additionally, the marginal association between cigarette affordability (as measured by PPP-adjusted prices) and reduced smoking frequency in males further corroborates the deterrent effect of higher prices on tobacco consumption. Price-based interventions, such as tax hikes, have been shown to effectively reduce youth smoking by making cigarettes less accessible. For instance, research shows that a 10% increase in cigarette prices reduces youth smoking initiation by 11.3%, highlighting the potential of taxation to counteract income-driven smoking behaviors [29]. However, while high-income countries included in our study often impose higher nominal cigarette prices, these products remain more affordable there compared to low- and middle-income countries (LMICs), where inflation tends to outpace price adjustments [30]. To effectively curb consumption, price increases must exceed both inflation rates and income growth. This underscores the importance of strategic pricing policies. Indeed, the WHO recognizes raising tobacco prices as the single most effective intervention to reduce smoking initiation, promote cessation, and ultimately decrease tobacco-related mortality [31].

Parental smoking emerged as a nuanced factor that differed by the sex of the adolescent. For males, paternal smoking was a significant predictor of higher smoking frequency, whereas maternal smoking surprisingly appeared to have a protective effect. In contrast, among females, maternal smoking—as well as the combined influence of both parents smoking—was associated with increased smoking frequency. This sex-differentiated pattern supports the hypothesis of same-sex parental role modeling and social learning theory, where children may be more likely to emulate the smoking behavior of the parent of the same sex [32–34]. Gender identity norms amplify this effect, as daughters internalize maternal smoking as socially acceptable behavior [34, 35]. Research has shown that maternal smoking influences both sexes but shows stronger associations with daughters [34, 36]. Contrary to the protective effect hypothesis, maternal smoking consistently elevates smoking risk across studies, though resilience factors (e.g., emotional regulation) can mitigate this impact [34, 37]. Paternal smoking, on the other hand, primarily predicts male smoking frequency, with no significant effect on daughters in most analyses [34, 35, 38]. Similar to our study, dual parental smoking increases smoking initiation odds and frequency, particularly for girls exposed to both parents’ tobacco use [34, 36]. Adolescents with engaged smoking parents show accelerated progression to regular use, suggesting observational learning amplifies nicotine’s biological effects [37]. Parental smoking cessation eliminates transmission risk, with former smokers’ children showing no increased likelihood of smoking [35, 36]. Contrary to some earlier studies that have emphasized peer influence as a critical factor in adolescent smoking initiation, our analyses revealed that, after adjusting for familial and individual factors, peer smoking did not significantly predict smoking frequency. This result suggests that the family environment, particularly parental behavior, may overshadow peer effects in determining how frequently adolescents smoke. Neuroscientific evidence suggests peer influence peaks during early adolescence due to heightened neural sensitivity to social rewards, but this may not translate to sustained smoking frequency without familial reinforcement [39, 40]. These findings have important implications for gender-targeted prevention strategies, parental smoking cessation programs, family-based intervention strategies, and resilience-building interventions to disrupt intergenerational tobacco use patterns.

It is important to note that while our analysis focuses on adolescents aged 13–15, the sex gap in smoking prevalence often reverses in later adolescence and early adulthood, with males surpassing females after age 18 [41, 42]. This shift may reflect biological and social factors, including later pubertal maturation in boys, evolving gender-role expectations, and gender-targeted marketing, that alter smoking trajectories as young people enter adulthood [41, 42]. Further longitudinal research is needed to elucidate how these puberty‐linked and sociocultural influences interact over the life course.

### Limitations

Despite its strengths; such as leveraging a large, cross-national dataset from the Global Youth Tobacco Survey and applying sophisticated multilevel modeling techniques, this study has several limitations. A key limitation is our exclusive reliance on recent GYTS data from eight relatively high-income European countries—spanning Central-Eastern to Southern Europe—which, while offering a current pan-European snapshot, may not generalize to regions without recent survey waves (e.g., Western and Northern Europe) or to low- and middle-income settings with different cultural norms, tobacco policies, and economic conditions. Another limitation stems from the method used to categorize pocket money into country-specific terciles (low, medium, high) based on national distributions. While this approach enables within-country comparisons, it may obscure true economic differences between countries. What is considered “high” pocket money in one country might correspond to “medium” or even “low” in another due to variations in the cost of living and overall income levels. This relative classification could lead to misestimations in the relationship between disposable income and smoking behavior, though the alignment of findings with previous research somewhat mitigates this concern. Additionally, while the analysis incorporated country-level factors such as PPP-adjusted cigarette prices and income inequality, other influential contextual variables—such as national tobacco control policies, cultural attitudes toward smoking, and enforcement of public health regulations—were not accounted for. The relatively high intraclass correlation coefficients (ICCs) at both the country and school levels further suggest that unmeasured contextual influences may play a significant role. Moreover, although standardized sampling weights enhance generalizability, variations in data collection methods across countries could have influenced the observed associations. Despite adjusting for several key individual and familial factors, residual confounding remains a possibility, as certain variables—such as nuanced parental behaviors, more detailed peer influences, and neighborhood characteristics—were not fully explored. The reliance on self-reported data introduces the potential for recall and social desirability biases, which may have affected the accuracy of reported smoking behaviors and parental smoking status. These biases could lead to either an underestimation or an overestimation of the true relationships between the predictors and smoking frequency. Additionally, while our three-level models account for clustering at the school level, we did not include any explicit school‐level covariates (e.g., school socioeconomic composition, tobacco policy enforcement, urban/rural status). As a result, we cannot distinguish whether variation in smoking frequency across schools is driven by contextual factors such as school‐based prevention programs or peer networks. Future studies should incorporate school‐level characteristics to more fully capture the influence of educational settings on adolescent smoking behaviors. Finally, given the cross‐sectional design, all associations reported herein are correlational and should not be interpreted as evidence of causality. We cannot exclude reverse causation or residual confounding affecting the observed relationships.

## Conclusion

In summary, our study highlights the critical roles of early smoking initiation and parental smoking in predicting smoking frequency among European adolescents. The sex-specific patterns observed underscore the need for tailored public health interventions that address both individual and familial risk factors. Building on these findings, future research should explore longitudinal trajectories of smoking behavior among adolescents, with an emphasis on how early initiation and familial influences evolve over time. Intervention studies that test the effectiveness of parental smoking cessation programs and economic measures (e.g., tax increases on tobacco products) would provide valuable insights into potential policy solutions. Additionally, qualitative studies could elucidate the psychosocial mechanisms through which parental smoking differentially affects boys and girls, thereby refining our understanding of sex-specific vulnerabilities in tobacco use.

## Data Availability

The GYTS datasets are publicly available data and are available on WHO NCD Microdata Repository through the following link: https://extranet.who.int/ncdsmicrodata/index.php/catalog/GYTS.
